# Continuous Isotropic-Nematic Transition in Amyloid Fibril Suspensions Driven by Thermophoresis

**DOI:** 10.1038/s41598-017-01287-1

**Published:** 2017-04-27

**Authors:** Daniele Vigolo, Jianguo Zhao, Stephan Handschin, Xiaobao Cao, Andrew J. deMello, Raffaele Mezzenga

**Affiliations:** 1 Department of Chemistry and Applied Biosciences, Institute for Chemical and Bioengineering, ETH Zurich, Vladimir-Prelog-Weg 1, Zurich, 8093 Switzerland; 2 Department of Health Science & Technology, Institute of Food, Nutrition and Health, ETH Zurich, Schmelzbergstrasse 9, Zurich, 8092 Switzerland; 3 Department of Materials, ETH Zurich, Wolfgang-Pauli-Strasse 10, CH-8093 Zurich, Switzerland; 4 School of Chemical Engineering, University of Birmingham, Edgbaston, Birmingham, B15 2TT UK

## Abstract

The isotropic and nematic (*I* + *N*) coexistence for rod-like colloids is a signature of the first-order thermodynamics nature of this phase transition. However, in the case of amyloid fibrils, the biphasic region is too small to be experimentally detected, due to their extremely high aspect ratio. Herein, we study the thermophoretic behaviour of fluorescently labelled β-lactoglobulin amyloid fibrils by inducing a temperature gradient across a microfluidic channel. We discover that fibrils accumulate towards the hot side of the channel at the temperature range studied, thus presenting a negative Soret coefficient. By exploiting this thermophoretic behaviour, we show that it becomes possible to induce a continuous *I*-*N* transition with the *I* and *N* phases at the extremities of the channel, starting from an initially single *N* phase, by generating an appropriate concentration gradient along the width of the microchannel. Accordingly, we introduce a new methodology to control liquid crystal phase transitions in anisotropic colloidal suspensions. Because the induced order-order transitions are achieved under stationary conditions, this may have important implications in both applied colloidal science, such as in separation and fractionation of colloids, as well as in fundamental soft condensed matter, by widening the accessibility of target regions in the phase diagrams.

## Introduction

In the presence of a temperature gradient, a colloidal particle dispersed in solution is subjected to a force that is proportional to and directed along the gradient of temperature. This physical effect is known as thermophoresis and it is well described albeit not completely understood^1^. In particular, the behaviour of spherical colloidal particles^2–4^, solutions of surfactants^5, 6^, polymers^7^, DNA^8–10^ and proteins^11, 12^ in the presence of temperature gradients have been extensively studied in recent years, with thermophoresis showing great utility in selectively separating particles by accumulation at the cold or the hot side^13, 14^. Among these systems, the study of the behaviour of anisotropic colloidal particles in the presence of a temperature gradient is a fascinating field that has not yet been fully explored^15, 16^. Specifically, while it is well understood how the excluded volume of a solution of anisotropic particles depends on their shape, and how this controls the phase behaviour^17^, the same behaviour in the presence of a temperature gradient remains to be established, at least in its generality.

A peculiar class of anisotropic particles are amyloid fibrils, which can be formed both *in-vivo* and *in-vitro* via conversion of native proteins into β-sheet-rich fibrillar aggregates with a long non-branched morphology. In the last decades, amyloid fibrils have been extensively explored due to their relevance to a diversity of neurodegenerative disorders^18–20^. Moreover, a large number of non-disease-associated proteins have also been shown to form amyloid fibrils with great application potentials^21, 22^. For example, bovine β-lactoglobulin protein, abundant in milk and in particular in whey, a byproduct of cheese production, can form amyloid fibrils with high average aspect ratios (length-to-diameter >300 in average) by heat denaturation and aggregation at low pH, low ionic strength and high temperature^23^. By increasing the volume fraction, a suspension composed of amyloid fibrils undergoes a first-order phase transition from a randomly oriented isotropic (*I*) phase to an ordered nematic (*N*) phase, where fibrils are preferentially oriented along a common direction^24, 25^. This is because the gain in free volume is higher than the loss in orientational entropy at sufficiently high concentration^17^. This phase transition is expected to be discontinuous from a thermodynamic point of view, so an intermediate concentration window is expected where the fibril suspension undergoes a macro-phase separation with an obvious interface, exhibiting a diluted *I* phase coexisting with a concentrated *N* phase at equilibrium, with the width of the region being inversely proportional to the aspect ratio^17, 26–30^. Outside this region of co-existence, either *I* or *N* is observed. In amyloid fibril systems, the *I* + *N* coexistence has remained elusive, most likely because the extremely high aspect ratios make the coexistence regime vanishingly small^17, 24, 30, 31^. Indeed, the biphasic region has only been observed at equilibrium by adjusting the interaction potential (e.g. adding a depleting polymer to change the effective second virial coefficient) or under non-equilibrium conditions, by locally concentrating fibrils using freeze−thaw cycles^31, 32^. Thus, in amyloid fibrils, either *I* or *N* phases are individually observed. In the present study, we employ a thermophoretic force to allow the simultaneous observation of the *I* and *N* phases, by accumulating β-lactoglobulin fibrils to one side of a channel (and depleting them from the opposite side), taking full advantage of the small size of microfluidic channels to maximize the magnitude of the temperature gradient (∇T, as high as tens of degrees per mm). This is of fundamental importance as it affords the capability of inducing a liquid crystalline phase transition on any rod-like particle system without having to change the composition, the physico-chemical properties or the interaction potential of the fibrils suspension.

We investigated the thermophoretic behaviour of a β-lactoglobulin fibril suspension in a microchannel to identify the best conditions that yield a simultaneous observation of the *I* and *N* phases within the same channel. The latter is to be observed along a concentration gradient (∇*c*) induced by thermophoresis, spanning a range that covers the threshold volume fraction and therefore leads to a phase transition.

At steady-state, the concentration gradient can be described by

1 $$\nabla c=-c(1-c){S}_{T}\nabla T$$

where *c* is the initial concentration of the suspension and *S*_*T*_ is the Soret coefficient, defined as:

2 $${S}_{T}={D}_{T}/D$$

where *D* is the Brownian mass diffusion coefficient and *D*_*T*_ is the thermophoretic mobility, that, in analogy to electrophoresis, relates the thermophoretic velocity, *v*_*T*_, to the temperature gradient via

3 $${v}_{T}={D}_{T}\nabla T$$

It is therefore essential to first characterise the Soret coefficient of β-lactoglobulin fibrils to guide a careful estimation of the required conditions for phase separation.

The separation attained by thermophoresis, in the case of diluted colloidal suspensions, can be then approximated by:

4 $$\frac{{\rm{\Delta }}c}{c}\approx -{S}_{T}{\rm{\Delta }}T$$

where ∆*c* = *c*_*hot*_ − *c*_*cold*_ is the concentration difference between the hot and cold side of the channel, with *∆T* being the temperature difference.

## Results

### Temperature dependence of thermophoresis of β-lactoglobulin fibrils

**Figure 1:**
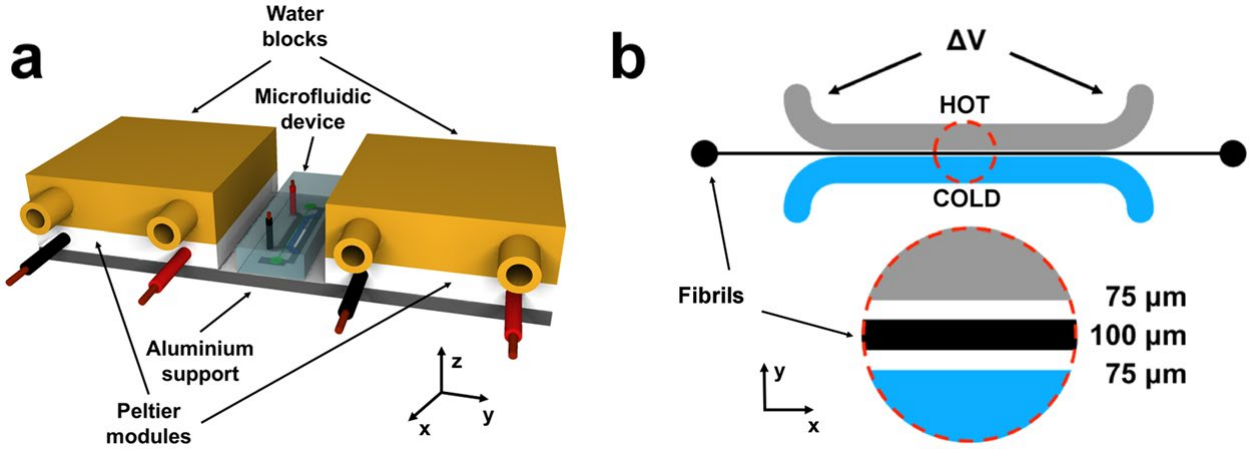
Schematic of the experimental setup for thermophoresis. (**a**) The microfluidic device is placed on an aluminium support, whose temperature is controlled by two separate Peltier modules and two water blocks are used as heat sink in conjunction with a temperature controlled water bath. (**b**) Detailed view of the microfluidic channel: a 30 mm long, 100 µm wide microchannel contains the fluorescent β-lactoglobulin fibrils, two larger (1 mm width) channels, separated by a 75 µm layer of polydimethylsiloxane (PDMS) from the microchannel, are used as heater (where ∆V is the potential applied to the Joule heater) and cooler to impose a temperature gradient. The whole device has a thickness of 120 µm.

Thermophoresis of aqueous suspensions depends (for a given system) on the average temperature at which the measurement is conducted^33^. We therefore performed a complete temperature dependence study of our system. To control the average temperature and to impose a precise temperature gradient across a microfluidic channel, we designed a bespoke experimental setup. As shown in Fig. 1, and described in details in the electronic supplementary information (ESI), we imposed and precisely controlled the average temperature by means of Peltier modules. We then used a previously described approach^13, 34^ to generate the required temperature gradient across the microchannel.

**Figure 2:**
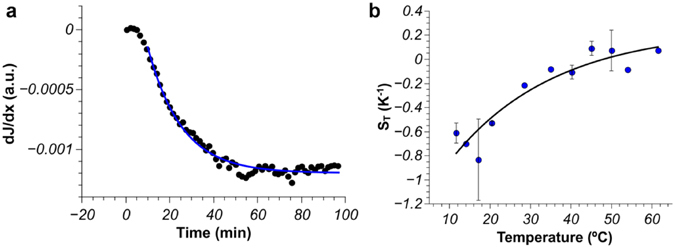
Thermophoretic behavior of β-lactoglobulin fibrils. (**a**) Typical gradient of the fluorescence intensity (*dJ*/*dx*) of fibrils measured across the microchannel during a thermophoretic experiment (c = 0.5% w/w, average T = 12 °C, ΔT = 14 °C/mm). The temperature gradient is imposed at time *t* = *0*. (**b**) Variation of the Soret coefficient, *S*_*T*_, with the average temperature. The solid line is a fit to the equation suggested by Iacopini and Piazza^33^.

Using fluorescent amyloid fibrils (0.5% w/w), we performed thermophoretic experiments and evaluated the concentration gradient under a temperature gradient by assessment of the fluorescence intensity *(J)* across the microfluidic channel as previously described^13, 34^. Briefly, we recorded a fluorescence image every 30 s and, by using a custom-made Matlab routine, we evaluated the fluorescence intensity across the channel. We then determined the slope of the linear intensity gradient that develops over time, *dJ/dx(t)*, which is directly proportional to the concentration gradient, *dc/dx(t)*. A representative measurement is shown in Fig. 2a, where the concentration profile reaches steady-state after 60 minutes. Knowledge of the initial volume fraction of the β-lactoglobulin fibrils, means that it is straightforward to convert the fluorescence intensity values into concentration, thus yielding the concentration gradient from each frame and the value of *S*_*T*_ at steady-state via eq. 1. It is worth to notice that the confinement of the sample in the microfluidic device guarantees that the convective effects are completely negligible.

We performed extensive experiments at different average temperatures between 12 and 60 °C to probe the dependence of the Soret coefficient. This variation closely follows the model proposed by Iacopini and Piazza^33^, i.e.

5 $${S}_{T}(T)={S}_{T}^{\infty }[1-\exp (\frac{{T}^{\ast }-T}{{T}_{0}})]$$

where ${S}_{T}^{\infty }$ represents the *S*_*T*_ limit for high temperature, *T* ^*^ is the characteristic temperature where *S*_*T*_ changes sign, and *T*_*0*_ is the exponential characteristic decay.

As shown in Fig. 2b, for an average temperature below 45 °C, we observe an accumulation of β-lactoglobulin fibrils towards the hot side, hence *S*_*T*_ < *0*. This can be explained by considering the fact that β-lactoglobulin fibrils are positively charged at pH 2 (as it is lower than the isoelectric point, pI = 5.2, for β-lactoglobulin)^35^. In the presence of a temperature gradient in acidic conditions (we use HCl to control the pH value), H^+^ ions tend to accumulate more strongly on the cold side than Cl^−^ ions^36, 37^. In other words, the specific Soret coefficient for H^+^ is larger than the one for Cl^−^ although both positive. Charge neutrality is conserved over the channel width, except at the boundaries where an excess of charge builds up, with the microchannel acting as a capacitor^5^; H^+^ ions will define a positive excess charge on the cold side, and Cl^−^ ions will accumulate at the hot side yielding a net negative charge. This situation gives rise to a thermoelectric field, i.e. an electric field due to the thermophoretic migration of the ions from the dissolved electrolytes^5, 38^. Since the ions are much smaller than β-lactoglobulin fibrils, the thermoelectric field builds up much faster than the β-lactoglobulin concentration gradient. As a result, β-lactoglobulin fibrils experience two forces: a pure thermophoretic force and a thermoelectric force. In the current experiments, we always evaluate the concentration gradient due to the total force acting on the β-lactoglobulin fibrils since the component contributions cannot be discriminated. Indeed, the thermoelectric force becomes apparent in a characteristic time much shorter than the typical time needed for β-lactoglobulin fibrils to drift appreciably because of the thermophoretic force (see ESI for more details). For an average temperature higher than 45 °C the total force acting on β-lactoglobulin fibrils becomes very small and as a consequence ${S}_{T}\approx 0$.

### Simultaneous presence of *I* and *N* phases across the channel induced by thermophoresis

**Figure 3:**
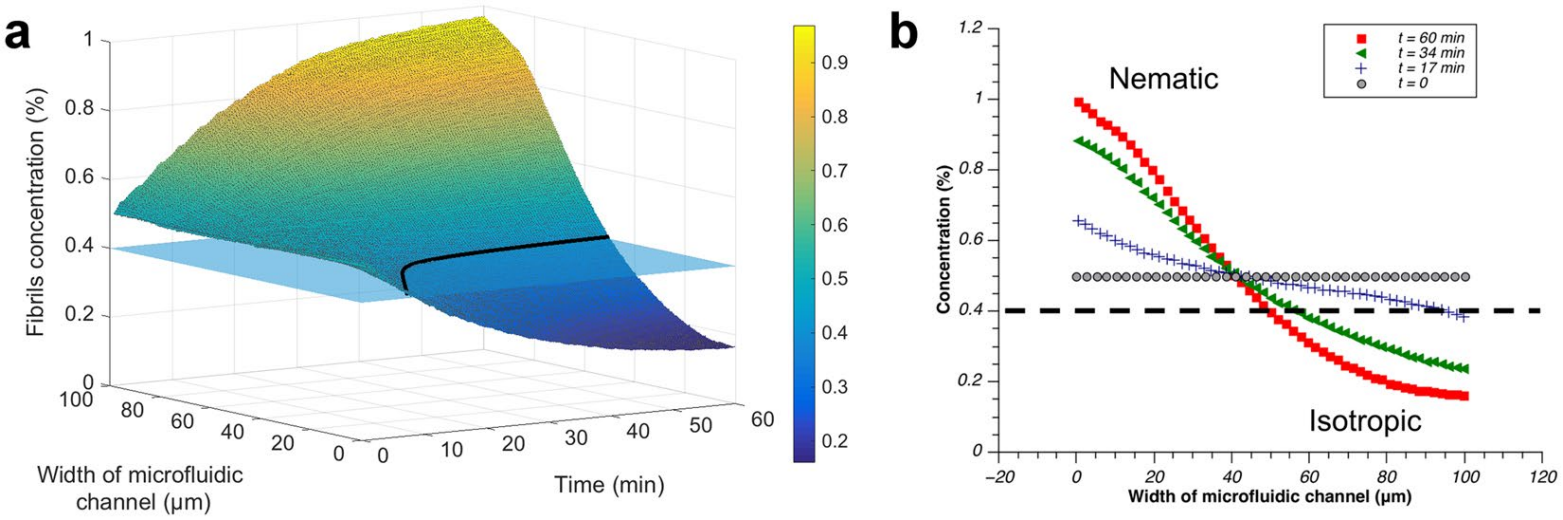
Temporal evolution of the concentration profile of a 0.5% w/w sample, at an average temperature of 17.8 °C in the presence of a temperature gradient of 20 °C/mm. (**a**) The colours represent local fibrils concentration, the plane shows the concentration at which the nominal transition between *I* and *N* under quiescent conditions is expected, and the black continuous line shows the corresponding position in the channel versus time, separating *I* and *N* phases under quiescent conditions. (**b**) Two-dimensional concentration profiles at different times. The black dashed line corresponds to the critical fibril concentration for the nominal *I*-*N* phase transition under quiescent conditions.

From the temperature dependence of the Soret coefficient we know that at low average temperatures the absolute value of the Soret coefficient, $|{S}_{T}|$, is larger, and thus for a fixed *∆T* the achievable separation will be higher (Fig. 2b). Accordingly, we decided to perform an experiment at a relatively low average temperature (17.8 °C) and large temperature gradient (20 °C/mm), as the concentration gradient induced by thermophoresis is directly proportional to $\nabla T$ (see eq. 1). Finally, we measured the temporal evolution of the concentration profile across the microchannel. As shown in the supplementary video, a remarkable unbalance in fluorescence intensity was achieved at steady-state, due to the accumulation of fluorescent β-lactoglobulin fibrils on the hot side of the microchannel induced by thermophoresis. Initially, the fibrils are homogeneously dispersed over the whole channel (Fig. 3). As time progresses, fibrils continue to accumulate on the hot side, with the concentration profile becoming gradually steeper. At steady state (after approximately 60 minutes) the β-lactoglobulin fibrils concentration becomes as high as 1% w/w on the hot side, while on the opposite (cold) side there is a depletion of β-lactoglobulin fibrils down to ≤0.2% w/w. Previous studies indicate that the critical concentration of the *I-N* phase transition is at ca 0.4% w/w^24^. Thus, the microchannel presents a biphasic region from a diluted *I* phase to a concentrated and ordered *N* phase along the width of the microchannel.

### Study of the birefringence of β-lactoglobulin fibrils under simultaneous *I* and *N* observation

**Figure 4:**
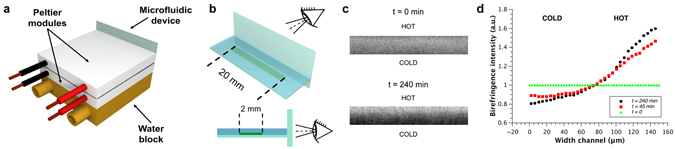
Direct observation of the *I* + *N* biphasic phase under microscopic polarized light. (**a**) Overall schematic of the cell used to record birefringence images and its temperature control through the use of two Peltier modules. (**b**) Enlargement of the microfluidic cell (not to scale). A thin glass cover slip placed perpendicular to the microfluidic cell permits optimized horizontal microscopic imaging under polarized light conditions; the thickness of the cell is 150 µm. (**c**) Birefringence that provides evidence of the simultaneous I and N phase presence at steady-state (t = 240 min) compared to initial conditions (t = 0) at which only *N* is present. Static spurious contributions coming from the PDMS device have been removed by applying a bandpass filter via FFT to the images in panel c via FIJI software. Nonetheless, a background signal is still present as evidenced by the fact that we still detect a non-zero signal on the cold (I phase) side of the channel (see Fig. 4d and Fig. S4). (**d**) Time evolution of the normalised birefringent intensity profile across the microchannel: from a uniform birefringent profile an I to N continuous profile appears over time.

Subsequently, we took advantage of the birefringence of the *N* phase in the fibrils suspension to provide direct evidence of the presence of the *I* and *N* phases across the channel at the same time. To observe a birefringence signal (c ≥ 0.4% w/w)^24^, we increased the optical length of the PDMS device to 2 mm, which is much longer than the one used for the fluorescence experiments previously described (120 µm). To prevent convection, we substantially modified the experimental setup by applying a vertical temperature gradient and heating the device from the top as this configuration minimises the Rayleigh–Bénard convection (Fig. 4). Moreover, to observe birefringence under these experimental conditions, the optical microscope was tilted by 90° and oriented horizontally (Fig. S3). We performed experiments on 0.5% w/w β-lactoglobulin fibrils at an average temperature of 18 °C and with a temperature gradient of approximately 23 °C/mm (Fig. S5), a condition similar to that described in Fig. 3. The results obtained under these conditions are shown in Fig. 4c. Under cross-polarized light, the suspension displays one single *N* phase at the initial stage (t = 0 min). At steady-state, after imposition of the temperature gradient (t = 240 min), a second phase is detected in the microchannel, presenting an intense birefringent *N* phase at the hot side where fibrils accumulate, and a dark *I* phase at the cold, fibril-depleted side. This provides direct evidence of phase separation from the originally single stable *N* phase into a system where *I* and *N* phases simultaneously exist within the same channel under a temperature gradient.

## Discussion

When comparing the present findings to the evolution of *I-N* phase separation in dispersions of anisotropic particles under gravity (over periods of days to months)^27–29^, the current kinetic process is significantly faster due to the presence of thermophoresis (which occurs within a few hours). Moreover, thermophoresis is well suited for liquid crystalline phase separation on the micron-scale, since it is easy to tune through variation of the magnitude of the temperature gradient and/or the average temperature. Finally, the degree of accumulation can be easily adjusted (Fig. 3), so that the volume fraction of the *N* phase and the birefringence intensity can be precisely regulated. Such experimental control is likely to have significant potential in the formation of functional films and microgels composed of ordered fibrous materials.

To conclude, we introduce a new strategy to control the phase diagram and associated order-order transitions of anisotropic colloidal suspensions, which may have far reaching implications in nanotechnology, materials and colloidal science. By imposing a temperature gradient along the width of a micro-fabricated channel, we observed thermophoretic migration of β-lactoglobulin amyloid fibrils toward the hot side. Due to the strong accumulation of fibrils at low average temperatures, the liquid crystal phase separation from one single *N* phase to a gradient of phases progressing continuously from the *I* to *N* phase was achieved. Interestingly, the results presented in Fig. 4 do not show a sharp change in intensity in neither the concentration nor the birefringence, as it would be expected for a first order thermodynamic transition. Thus, while the presence of an *I* and *N* phase is conclusive, our results do not allow ruling out a possible second order thermodynamic transition in this specific experimental conditions, for which a continuous transition of concentration and order parameter is to be expected. This could arise, among other things, from the gradient conditions imposed or from the presence of anchoring of the fibrils at the interfaces, and should be subject to further deeper investigation. Nonetheless, our results show, for the first time, that thermophoresis is a powerful tool for promoting liquid crystal phase transitions and for precisely controlling concentration/ordering gradients of fibrous systems on the micron scale, simply by adjusting operating temperatures. Since thermophoresis is particularly suitable for such measurements (i.e. it is a physical effect that does not require labelling or changes in chemical conditions and compositions of the samples) the present methodology shows significant promise in the fine control of order-order transitions in anisotropic colloidal dispersions.

## Materials and Methods

### Microfluidic devices fabrication

We fabricated all the microfluidic devices by standard soft-lithography technique^39^ in polydimethylsiloxane (PDMS). Briefly, a mould was obtained by spin coating a layer of a photoresist resin (SU-8, Microchem) on a 4″ silicon wafer (Si-Mat, Germany), and by exposing it to UV light through a high resolution photomasks (Micro Lithography Services Ltd, UK) that we designed using AutoCAD 2016 (Autodesk, USA). As a result, we obtained a negative mould on top of which we poured the PDMS mixture (Sylgard 184, Dow Corning, USA) in a base to curing agent ratio of 10:1. After curing, we punched inlet and outlet holes in the PDMS channels and we bonded them irreversibly on glass coverslips (24 mm × 60 mm, Menzer Glaser, USA) by corona discharge treatment (BD-20AC, Electro-Technic Products).

### Temperature control

We imposed a temperature gradient by creating an embedded Joule heater made from a low melting point alloy (MCP-96, 5 N Plus, Germany) as the high temperature source, and delivered cold water parallel to the sample channel. The temperature was measured using two thermocouples (Farnell, LABFACILITY, 200 µm diameter) placed in direct contact with the glass cover slip (170 μm thickness) below the heater and cooler on each side of the channel, and the temperature gradient across the microchannel accounted based on the temperature drop across the materials between the thermocouple and the sample (see ESI for more details).

### Purification of β-lactoglobulin protein from whey

Whey protein (Davisco, USA) was dissolved in Milli-Q water at 15% w/v, and stirred at 4 °C overnight. Afterwards, sodium citrate tribasic dihydrate (Sigma-Aldrich) and citric acid monohydrate (Fisher Scientific, USA) were mixed in the protein solution under vigorous stirring, to attain a citric concentration of 60 g/L and a pH value ~3.6. Then, the mixture was heated at 50 °C for 2h using a water bath, and incubated at room temperature for 24 h. After a slow (4 000 rpm, 20 min) and following fast centrifugation (14 000 rpm, 20 min) at room temperature, a nearly transparent solution and white sediment were obtained. The supernatant was collected and the heat-precipitation-centrifugation procedure was repeated for two more consecutive times before filtration by means of a 0.45 µm cellulose acetate membrane filter (Sigma-Aldrich). The filtered protein solution was dialyzed using a Spectra/Por dialysis membrane with 6–8 kDa MWCO (Spectrum Laboratories) against Milli-Q water at 4 °C, which was previously boiled for 10 min in 1 mM EDTA solution and extensively rinsed. Finally, the dialyzed solution was freeze-dried and kept at −20 °C for long-term storage.

### Synthesis of β-lactoglobulin amyloid fibrils

#### Fluorescent fibrils

2 mM of Thiazole orange (TO, Sigma-Aldrich) solution was prepared by dissolving the appropriate amount of dye in dimethyl sulfoxide (DMSO, Sigma-Aldrich). Then, the mixture of 2% w/w purified β-lactoglobulin protein and 60 µM TO was prepared in Milli-Q water and adjusted to pH 2 using 1 M HCl solution. The solution was filtered through a 0.2 µm syringe filter with cellulose acetate membrane (VWR). After a heat treatment at pH 2 and 90 °C for 5 h, the sample was cooled down immediately using water-ice mixture. The obtained fluorescent fibrils suspension was centrifuged at 3000 rpm and 20 °C for 20 min and then stored at 4 °C, used for the experiments within one week.

#### Non-fluorescent fibrils

The procedure was similar to that of fluorescent fibrils, but no TO solution was added.

### Microscopy experiments and setup

The fluorescence microscopy experiments were conducted on an inverted microscope (Ti-E, Nikon, Switzerland) equipped with a multicolour LED light source (Lumencor Spectra), and a highly sensitive camera (Hamamatsu ORCA4.0 v2) controlled by an open-source software (Micro-Manager^40, 41^), while the birefringent experiments were performed on a AxioScope (Zeiss) light microscope tilted by 90° equipped for polarized light microscopy (see ESI for more details). The collected fluorescence images where then analysed using a custom Matlab routine in order to extract the concentration gradient induced by thermophoresis and evaluate the corresponding Soret coefficient as described in detail in the ESI.

### Numerical simulations of the temperature distribution inside the microchannel

Two-dimensional numerical simulations were performed using Comsol Multiphysics 4.4 to determine the temperature distribution across the microchannel for different experimental conditions. An extra fine mesh was used to obtain steady-state temperature distribution across the different materials composing the device modelled based on the Comsol’s materials library (more details in the ESI).

## Electronic supplementary material

supplementary information

supplementary video

## References

[1] Piazza R, Parola A (2008). Thermophoresis in colloidal suspensions. J. Phys. Condens. Matter.

[2] Braibanti M, Vigolo D, Piazza R (2008). Does thermophoretic mobility depend on particle size?. Phys. Rev. Lett..

[3] Putnam SA, Cahill DG (2005). Transport of nanoscale latex spheres in a temperature gradient. Langmuir.

[4] Duhr S, Braun D (2006). Thermophoretic Depletion Follows Boltzmann Distribution. Phys. Rev. Lett..

[5] Vigolo D, Buzzaccaro S, Piazza R (2010). Thermophoresis and thermoelectricity in surfactant solutions. Langmuir.

[6] Piazza R, Guarino A (2002). Soret Effect in Interacting Micellar Solutions. Phys. Rev. Lett..

[7] Rauch J, Köhler W (2005). On the molar mass dependence of the thermal diffusion coefficient of polymer solutions. Macromolecules.

[8] Duhr S, Arduini S, Braun D (2004). Thermophoresis of DNA determined by microfluidic fluorescence. Eur. Phys. J. E. Soft Matter.

[9] Reineck P, Wienken CJ, Braun D (2010). Thermophoresis of single stranded DNA. Electrophoresis.

[10] Morasch M, Braun D, Mast CB (2016). Heat-Flow-Driven Oligonucleotide Gelation Separates Single-Base Differences. Angew. Chemie Int. Ed..

[11] Iacopini S, Piazza R (2003). Thermophoresis in protein solutions. Europhys. Lett..

[12] Wolff M (2016). Quantitative thermophoretic study of disease-related protein aggregates. Sci. Rep.

[13] Vigolo D, Rusconi R, Stone HA, Piazza R (2010). Thermophoresis: microfluidics characterization and separation. Soft Matter.

[14] Geelhoed PF, Lindken R, Westerweel J (2006). Thermophoretic Separation in Microfluidics. Chem. Eng. Res. Des..

[15] Garcia‐Ybarra P, Rosner D (1989). Thermophoretic properties of nonspherical particles and large molecules. AIChE J..

[16] Wang Z, Kriegs H, Buitenhuis J, Dhont JKG, Wiegand S (2013). Thermophoresis of charged colloidal rods. Soft Matter.

[17] Onsager L (1949). The effects of shape on the interaction of colloidal particles. Ann. N. Y. Acad. Sci.

[18] Selkoe DJ (2003). Folding proteins in fatal ways. Nature.

[19] Chiti F, Dobson CM (2006). Protein Misfolding, Functional Amyloid, and Human Disease. Annu. Rev. Biochem..

[20] Hamley IW (2007). Peptide Fibrillization. Angew. Chemie Int. Ed..

[21] Cherny I, Gazit E (2008). Amyloids: Not Only Pathological Agents but Also Ordered Nanomaterials. Angew. Chemie Int. Ed..

[22] Li C, Mezzenga R (2013). The interplay between carbon nanomaterials and amyloid fibrils in bio-nanotechnology. Nanoscale.

[23] Adamcik J (2010). Understanding amyloid aggregation by statistical analysis of atomic force microscopy images. Nat. Nanotechnol.

[24] Jung J-M, Mezzenga R (2010). Liquid crystalline phase behavior of protein fibers in water: experiments versus theory. Langmuir.

[25] Corrigan AM, Müller C, Krebs MRH (2006). The Formation of Nematic Liquid Crystal Phases by Hen Lysozyme Amyloid Fibrils. J. Am. Chem. Soc..

[26] Flory PJ (1956). Phase Equilibria in Solutions of Rod-Like Particles. Proc. R. Soc. A Math. Phys. Eng. Sci.

[27] Buining PA, Lekkerkerker HNW (1993). Isotropic-nematic phase separation of a dispersion of organophilic boehmite rods. J. Phys. Chem..

[28] Buining PA, Philipse AP, Lekkerkerker HNW (1994). Phase Behavior of Aqueous Dispersions of Colloidal Boehmite Rods. Langmuir.

[29] Van Bruggen MPB, Dhont JKG, Lekkerkerker HNW (1999). Morphology and kinetics of the isotropic-nematic phase transition in dispersions of hard rods. Macromolecules.

[30] Viamontes J, Oakes PW, Tang JX (2006). Isotropic to Nematic Liquid Crystalline Phase Transition of F -Actin Varies from Continuous to First Order. Phys. Rev. Lett..

[31] Zhao J (2016). Freeze–Thaw Cycling Induced Isotropic–Nematic Coexistence of Amyloid Fibrils Suspensions. Langmuir.

[32] Zhao J, Li C, Mezzenga R (2014). Re-entrant isotropic-nematic phase behavior in polymer-depleted amyloid fibrils. J. Phys. Condens. Matter.

[33] Iacopini S, Rusconi R, Piazza R (2006). The “macromolecular tourist”: Universal temperature dependence of thermal diffusion in aqueous colloidal suspensions. Eur. Phys. J. E.

[34] Vigolo D, Rusconi R, Piazza R, Stone HA (2010). A portable device for temperature control along microchannels. Lab Chip.

[35] Jones OG (2011). Complexation of β-Lactoglobulin Fibrils and Sulfated Polysaccharides. Biomacromolecules.

[36] Agar JN, Turner JCR (1960). Thermal Diffusion in Solutions of Electrolytes. Proc. R. Soc. A Math. Phys. Eng. Sci.

[37] Agar JN, Mou CY, Lin JL (1989). Single-ion heat of transport in electrolyte solutions: a hydrodynamic theory. J. Phys. Chem..

[38] Würger A (2008). Transport in Charged Colloids Driven by Thermoelectricity. Phys. Rev. Lett..

[39] Duffy DC, McDonald JC, Schueller OJ, Whitesides GM (1998). Rapid Prototyping of Microfluidic Systems in Poly(dimethylsiloxane). Anal. Chem..

[40] Edelstein, A., Amodaj, N., Hoover, K., Vale, R. & Stuurman, N. In *Current Protocols in Molecular Biology* **921420**, (John Wiley & Sons, Inc., 2010). 10.1002/0471142727.mb1420s92 PMC3065365 20890901

[41] Edelstein AD (2014). Advanced methods of microscope control using μManager software. J. Biol. Methods.

