# Supplementary material for: Continuous Isotropic-Nematic Transition in Amyloid Fibril Suspensions Driven by Thermophoresis
Source: Sci Rep. 2017 Apr 27;7:1211. doi: 10.1038/s41598-017-01287-1 (PMC5430637; doi:10.1038/s41598-017-01287-1)
Supplement: Supplementary file 1 — supplementary information [file 41598_2017_1287_MOESM1_ESM.pdf]

Electronic Supplementary Information (ESI) for

Continuous Isotropic-Nematic Transition in Amyloid Fibril Suspensions Driven by Thermophoresis

Daniele Vigolo[†, ‡, 1], Jianguo Zhao[†, 2], Stephan Handschin[2], Xiaobao Cao[1], Andrew J. deMello[1] and Raffaele Mezzenga[2, 3]*

[†] Equal contribution.

* Corresponding author: raffaele.mezzenga@hest.ethz.ch.

[1] Department of Chemistry and Applied Biosciences, Institute for Chemical and Bioengineering, ETH Zurich, Vladimir-Prelog-Weg 1, Zurich 8093, Switzerland.

[2] Department of Health Science & Technology, Institute of Food, Nutrition and Health, ETH Zurich, Schmelzbergstrasse 9, Zurich 8092, Switzerland.

[3]Department of Materials, ETH Zurich, Wolfgang-Pauli-Strasse 10, CH-8093 Zurich, Switzerland.

‡ Present address: School of Chemical Engineering, University of Birmingham, Edgbaston, Birmingham, B15 2TT, UK.

Purity analysis of β-lactoglobulin protein from whey.

After the heat induced selective aggregation and precipitation, highly purified β-lactoglobulin protein was obtained, where no α-lactalbumin or other protein was detected (see FIG. S1).

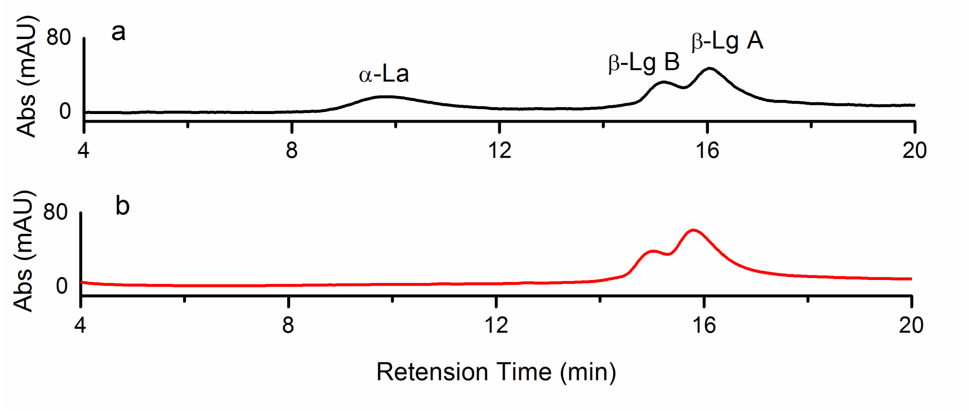

FIG.S1 HPLC chromatograms of protein solutions. a. native whey, b. purified β-lactoglobulin.

Fluorescence of TO bound amyloid fibrils.

TO dye was chosen for its capability to maintain the fluorescent emission at low pH and strong fluorescence intensity after aggregation with amyloid fibrils[1]. We evaluate the absorbance and emission spectrum of the labelled β-lactoglobulin fibrils (2 % w/w of β-lactoglobulin and 60 μM TO) and, as a control, the same concentration of dye in solution. As shown in FIG. S2a we obtained three absorbance peaks (around 422 nm, 501 nm and 548 nm, respectively) for the TO dye and fluorescent β-lactoglobulin fibrils. Once excited at 422 nm the labelled β-lactoglobulin fibrils strongly emit at about 462 nm while the emission of the dye is completely negligible (see FIG. S2b), confirming that the chosen dye only emits once bind. This result also guarantees that the fluorescent signal recorded is solely dependent on the fluorescent fibrils.

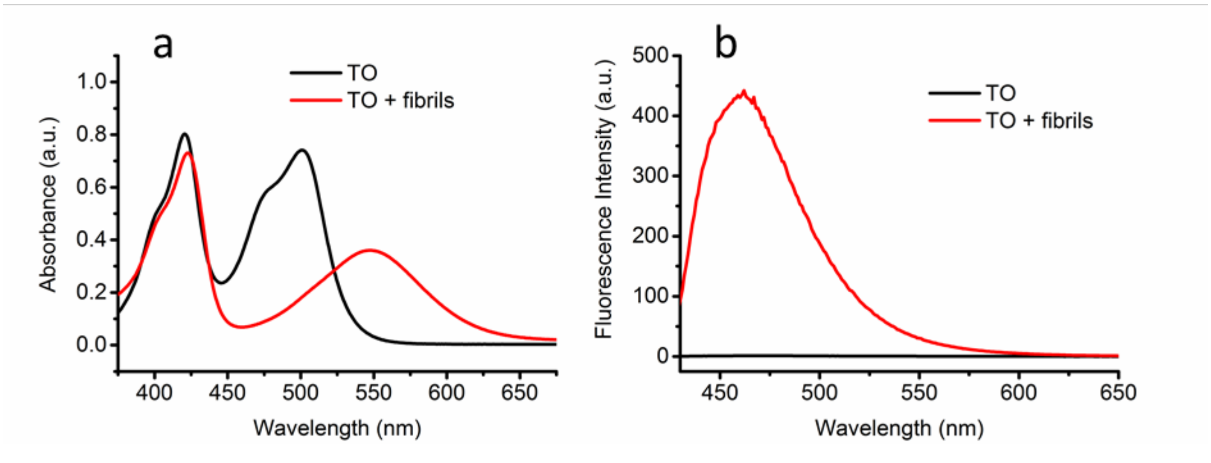

FIG. S2. a. Absorption spectra of fluorescent BLG fibrils and free TO dye. b. Emission spectra of fluorescent BLG fibrils and free TO dye when excited at 422 nm.

Temperature control of the experimental setup.

As mentioned in the main text, we imposed an average temperature by controlling two independent Peltier modules connected to two water blocks in which we circulated water at a fixed temperature to improve the heat exchange. We monitored their temperature via the software Signal express 2014 (National Instruments) using two thermocouples (Farnell, Lab facility, 200 μm diameter) placed in direct contact with the Peltier modules interfaced to a computer via a data acquisition board (National Instrument, NI 9211). The Peltier modules and the water blocks were then attached to the bottom side of an aluminium support. A slit in the middle to allowed optical access to the microfluidic device that was placed on top of the support (see FIG. 1a). The generation of the temperature gradient across the microfluidic

channel (100 μm width, 120 μm thickness), was established by designing two large channels (1 mm width, 120 μm thickness) one at each side of the microchannel containing the sample solution as shown in FIG. 1b. This is a similar approach to what we described in[2,3] where we used one of this large channel filled with a conductive epoxy resin as a Joule heater. In the present paper we used instead a low melting point alloy (MCP-96, obtained as free sample by 5N Plus, Germany) that was made to flow by raising its temperature on a hot plate (melting temperature 96 °C) and solidified at room temperature. This novel approach permitted to impose a very stable and uniform temperature across the whole length of the Joule heater. The second large channel was used as the cold side of the temperature gradient by flowing cold water at a high flow rate.

We then evaluated the temperature drop across the PDMS layer between the heater (and cooler) and the sample channel. Knowing the thermal conductivities, k, of the materials involved (we used k_{PDMS} = 0.15 W/mK and, for the aqueous sample, k_{water} = 0.6 W/mK), it is easily possible to obtain the actual ΔT across the sample's channel. In fact:

$$\Delta T_{sample} = \Delta T_{measured} \, {}^{R_{sample}}\!/_{R_{tot}} \qquad \text{(S1)}$$

Where ΔT_{sample} is the actual temperature across the sample's channel, $\Delta T_{measured}$ is the temperature difference measured by the thermocouples, and with R we define the thermal resistivity in the electronic analogy. In particular $R_{sample} = {}^{d_{sample}}\!/_{k_{water}}$ and $R_{tot} = 2\,{}^{d_{PDMS}}\!/_{k_{PDMS}} + {}^{d_{sample}}\!/_{k_{water}}$, where d_{sample} and d_{PDMS} are respectively the width of the sample channel and the PDMS wall.

Evaluation of Soret coefficient (S_T).

Using a highly sensitive fluorescent camera (Hamamatsu ORCA4.0 v2) controlled by the Micro-Manager open source software[4,5], we collected a fluorescent image every 30 s. Then, using a custom Matlab routine, each frame was normalised using the first frame acquired without temperature gradient. In this way random imperfections in the fluorescence distribution due to static defects in the device are eliminated. Also, the intensity of each frame is normalised to have the same average value of the first frame; this is done to compensate for quenching of the dye and it simply reflects mass conservation (fluorescence intensity is in fact proportional to the mean volume fraction which is not changing during the experiment). Finally, each frame intensity is averaged along the channel length (i.e. perpendicular to the temperature gradient) in order to obtain a single intensity profile from each frame. The concentration gradient developed inside the microfluidic channel is linear at the centre of the channel, for this reason we fit the intensity profile with a linear interpolation curve over about the central 80% width of the profile and we extract the slope, *dJ/dx*, which is directly proportional to the concentration gradient, *dc/dx*. Knowing the initial volume fraction (in our case 0.5% w/w) of the β-lactoglobulin fibrils, we converted the fluorescence intensity values into concentration, thus obtaining concentration gradient information from each frame. By plotting the value of *dJ/dx* versus time we were also able to follow the dynamics of the build-up of the concentration gradient induced by thermophoresis (see FIG. 2a in the main text) which follows an exponential decay. From the fitting curve we obtained the characteristic time, τ, and the plateau value (for $t \rightarrow \infty$) that corresponds to the steady state concentration gradient. The parameter τ is directly related to the Brownian diffusion coefficient, *D*, through the well-known relationship:

$$D = w^2/\pi^2\tau \qquad \text{(S2)}$$

where w = 100 μm is the channel width. The steady state value of ∇c is then related to the Soret coefficient via:

$$\nabla c = -c(1-c)S_T\nabla T \qquad \text{(S3)}$$

as discussed in the main text.

The overall drift detected is the result of the balance between the thermophoretic force acting on the fibrils and the thermoelectric force due to the thermophoretic drift of the ions dispersed in solution as described in the main text and in[6,7]. These forces act on different characteristic time scale and the difference depends on the characteristic mass diffusion time, τ, of ions (responsible for the thermoelectric force) and β-lactoglobulin fibrils in the presence of a temperature gradient, which is related to their hydrodynamics radii. In particular, $\tau = w^2/\pi^2 D$, where w is the width of the microchannel and D is the Brownian diffusion coefficient defined by the Stokes-Einstein relation as: $D = k_B T/6\pi\eta R$, where k_B is the Boltzmann constant, η the viscosity and R the hydrodynamic radius. As a result, the thermoelectric field builds up within the channel approximately 1000 times faster than the thermophoretic drift. It is thus impossible to evaluate these two contributions separately and we always refer to the total force instead.

Detection of birefringence signal.

Birefringence is directly proportional to the optical path along which we are illuminating the sample. In particular, for thin samples size (of the order of 0.2 mm) for which the optical path was shorter than the optimum, the signal was too weak to be detected, while for longer optical path (several mm) difficulties in alignment made impossible to obtain a reliable signal. We performed a series of tests to calibrate our optical system and to optimize the width of the sample channel to reliably detect the birefringence signal. The optimal length was determined to be 2 mm. For such a long optical path the temperature gradient cannot be imposed horizontally as the unavoidable Rayleigh–Bénard convection will be significant as described for example by Rusconi et al.[8]. As a consequence, no flow is present within the microfluidic device except from the thermophoretic drift of the fibrils under investigation, thus no flow-induced birefringence effect is detected.

To apply a vertical (i.e. parallel to gravity) temperature gradient, we used two Peltier modules coupled to a water block for the cold side to increase its efficiency, while we kept the Peltier used for the hot side to work against the ambient air as shown in FIG. 4a. The microfluidic channel was then placed in between the two Peltier modules. The microfluidic chip was composed by a 2 mm wide channel (the optical path over which we measured the birefringence signal), 150 μm thickness and about 20 mm long; it was made of thin PDMS (the overall thickness of the PDMS device was about 500 μm) attached to a thin glass cover slip (170 μm) by corona discharge treatment. To be able to have optical access to the side of the channel, a second glass cover slip was attached perpendicular to the side of the channel by coating it with a thin layer of PDMS (about 250 μm) using a spin coater (500 rpm for 30 seconds), following by a curing period of about 2 hours in oven at 70 °C. In this way we obtained a thin device where to apply a vertical temperature gradient while maintaining optical access along the 2 mm long side of the channel. The whole setup was then placed in the light path of a AxioScope (Zeiss) light microscope tilted by 90° (see FIG. S3) and set up for polarized light microscopy. We controlled the temperature of the two Peltier modules and, by knowing the thermal characteristics of the materials used, estimated the actual temperature difference inside the microchannel (as discussed in the section "Temperature control"). Images were recorded every minute for the first 20 minutes and every 10 minutes subsequently, and then analysed with a similar Matlab routine as described in the S_T measurement. The main difference from the previously described methos is that we are not normalising the intensity of each frame, because birefringence is not subjected to quenching as fluorescence and it is not measurable when the fibrils concentration is below the *I-N* threshold concentration. In addition, we are not expecting a linear gradient of intensity. In fact, we expected to measure a birefringence signal for the sample without temperature gradient (as the concentration, c = 0.5% w/w is in nematic phase), and a subsequent decreased signal towards the cold side (where the local concentration of fibrils decreases due to the thermophoretic drift) and an increased signal towards the hot side (where fibrils accumulate). As a result, at steady state, we expected a non-linear profile, with a dark region towards the cold side (isotropic phase) and a bright one towards the hot side (nematic) as shown in FIG. 4c in the main text.

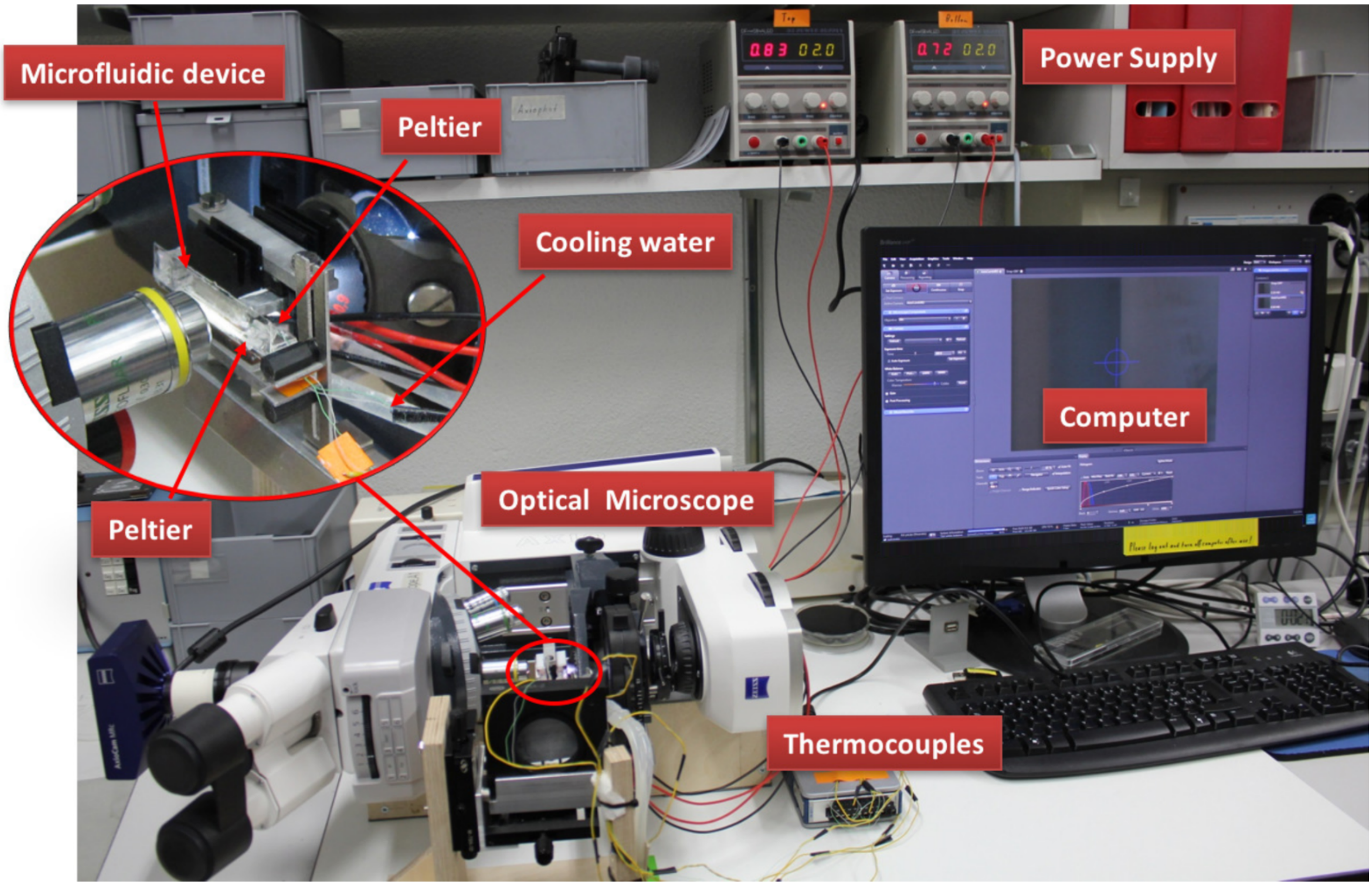

FIG. S3: Experimental setup for birefringence observation of amyloid fibrils suspension under microscopic polarized light in the presence of vertical temperature gradient.

Analyses of the birefringent signal.

In order to calibrate our birefringent signal, we performed a series of tests for different optical length (0.5, 1 and 2 mm), for different fibrils concentrations and acquiring images at different exposure time to guarantee reliability of the data. It is important to notice that in our setup we always have a background birefringent signal coming from the PDMS device and we took this into account by comparing the signal obtained with the one of pure deionised water. We present a summary of these experiments in FIG. S4 where we show the comparison in birefringence signal between water and two different concentration of β-lactoglobulin fibrils (0.5% and 1%) acquired at 5 different exposure times for an optical path of 2 mm. In FIG. S4d we compared the relative intensity of each case. In our experiments, we fixed the exposure time to 100 ms in order to guarantee a better discrimination of the signal coming from the β-lactoglobulin fibrils.

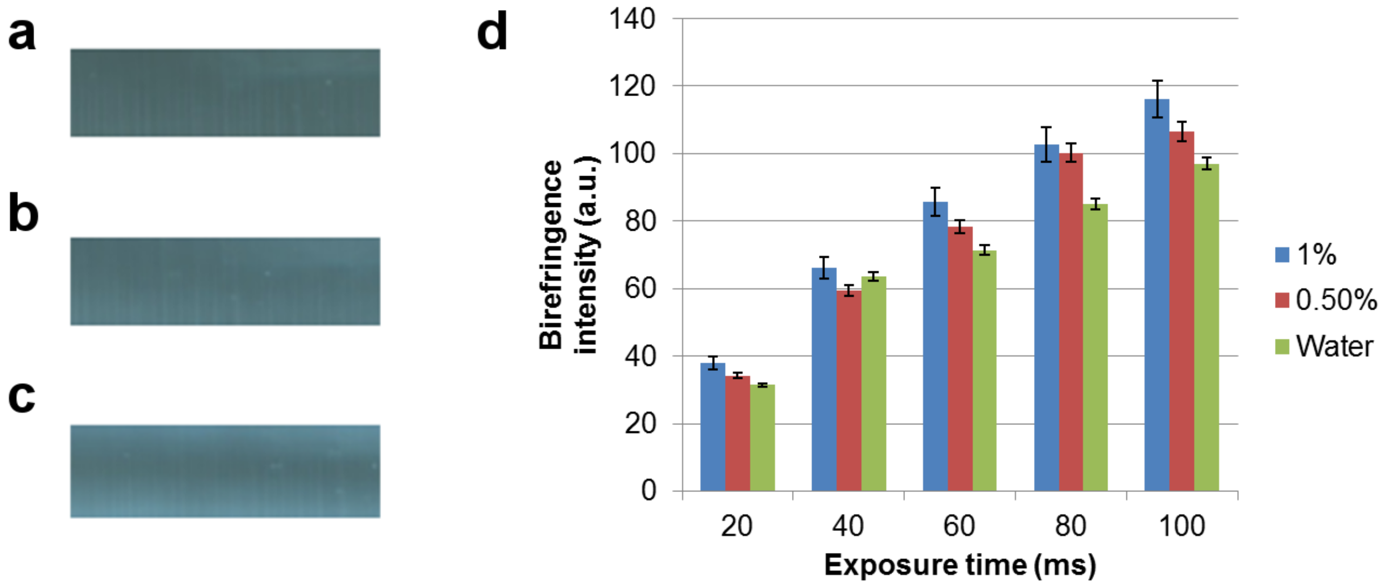

FIG. S4. Analyses of the birefringent signal. a – c: unprocessed birefringent images obtained for, respectively, deionised water, 0.5% and 1% β-lactoglobulin fibrils solutions for a device with a 2 mm optical path and for an exposure time of 100 ms. d. average birefringent signal intensity of deionised water, 0.5% and 1% β-lactoglobulin fibrils solutions for a device with a 2 mm optical path and for an exposure time of 20, 40, 60, 80 and 100 ms. The error bars represent the standard deviation obtained from the average of the intensity of the recorded images for each sample. The residual birefringence of water, which is used as a control, is caused by the PDMS.

Numerical simulation: temperature distribution across the channel.

We performed 2D numerical simulations using Comsol Multiphysics 4.4 to determine the temperature distribution within the microchannel for different experimental conditions. We reproduced the geometry and the dimensions of our experimental setup, and we used the built in library for the choice of the materials properties. In order to determine the temperature gradient inside the channel, we impose a uniform temperature as measured by the thermocouples at the hot and cold side of the heater and cooler used (either the embedded ones used for the temperature dependence experiments, or the Peltier modules as used for the birefringence experiments). We then let Comsol determine the temperature drop across each material and we evaluated the final temperature distribution across the channel filled by β-lactoglobulin fibrils suspension. In the case of birefringence experiment (see FIG. S5), the actual temperature gradient across the sample is as big as tens of degrees per millimetre due to the small size of the microchannel (150 μm).

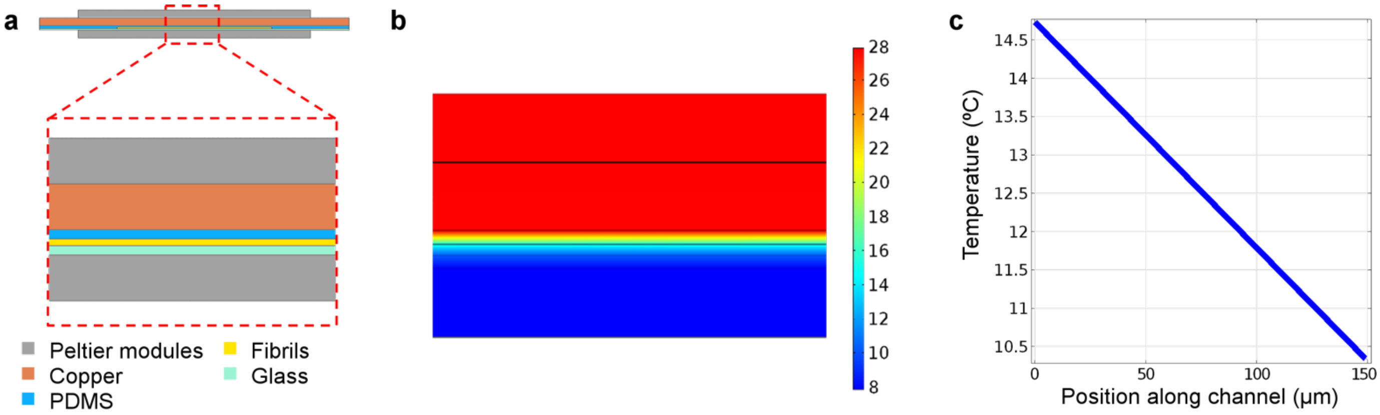

FIG. S5. Numerical simulations of the temperature distribution inside the PDMS device used for birefringence experiments. a. Cross section of the microfluidic device with a zoom-in view. To note, the copper layer is used to guarantee a better heat transfer from the Peltier module to the thin PDMS layer. b. Temperature distribution across the device in the case of T_{HOT} = 28 °C and T_{COLD} = 8 °C. c. Temperature across the microchannel containing the β-lactoglobulin fibrils: it is possible to appreciate how the actual temperature of sample changes as a function of channel position.

Supporting Video Legend

A fluorescently labelled sample of β-lactoglobulin fibrils (c = 1% w/w) in a microfluidic channel (100 µm wide and 120 µm thick) at a relatively low average temperature (17.8 °C) in the presence of a temperature gradient (20 °C/mm). The hot side is on top and the cold side on the bottom of the microfluidic channel. It is possible to appreciate how, over time, the fibrils migrate towards the hot side creating a concentration gradient as shown by the increased fluorescent intensity at the hot side. From the intensity profile of each image is possible to quantify the concentration gradient and evaluate the Soret coefficient as discussed in the main text and shown in Fig. 2. The images are collected every 30s and the fluorescent intensity is normalised to account for possible quenching of the dye.

References

1. Gao, G.-Q. & Xu, A.-W. A new fluorescent probe for monitoring amyloid fibrillation with high sensitivity and reliability. *RSC Adv.* **3,** 21092 (2013).

2. Vigolo, D., Rusconi, R., Piazza, R. & Stone, H. A. A portable device for temperature control along microchannels. *Lab Chip* **10,** 795–8 (2010).

3. Vigolo, D., Rusconi, R., Stone, H. A. & Piazza, R. Thermophoresis: microfluidics characterization and separation. *Soft Matter* **6,** 3489 (2010).

4. Edelstein, A., Amodaj, N., Hoover, K., Vale, R. & Stuurman, N. in *Current Protocols in Molecular Biology* **921420,** (John Wiley & Sons, Inc., 2010).

5. Edelstein, A. D. *et al.* Advanced methods of microscope control using µManager software. *J. Biol. Methods* **1,** 10 (2014).

6. Vigolo, D., Buzzaccaro, S. & Piazza, R. Thermophoresis and thermoelectricity in surfactant solutions. *Langmuir* **26,** 7792–801 (2010).

7. Würger, A. Transport in Charged Colloids Driven by Thermoelectricity. *Phys. Rev. Lett.* **101,** 108302 (2008).

8. Rusconi, R., Isa, L. & Piazza, R. Thermal-lensing measurement of particle thermophoresis in aqueous dispersions. *J. Opt. Soc. Am. B* **21,** 605 (2004).
